# Prevention of the Aggregation of Nanoparticles during the Synthesis of Nanogold-Containing Silica Aerogels

**DOI:** 10.3390/gels4020055

**Published:** 2018-06-19

**Authors:** István Lázár, Hanna Judit Szabó

**Affiliations:** Department of Inorganic and Analytical Chemsitry, University of Debrecen, Egyetem tér 1, H-4032 Debrecen, Hungary; szabohanna96@gmail.com

**Keywords:** silica aerogel, nanogold, AuNP, nanocomposite, induced aggregation, plasmonic aerogel, sol-gel process, plasmon resonance

## Abstract

Nanogold is widely used in many areas of physics and chemistry due to its environment-sensitive plasmon resonance absorption. The immobilization of gold nanoparticles in highly porous silica aerogel offers an attractive alternative to liquid gold solutions as they show a mechanically stable structure, are permeable to gases, and can even be used at elevated temperatures. We have found that the commercially available citrate-stabilized 10 nm gold nanoparticles may suffer from aggregation prior to or under the base-catalyzed gelation process of tetramethoxy silane. In the wet gels, Au particles increased in size, changed shape, and demonstrated the loss of plasmon resonance absorption, due to the formation of larger aggregates. We have studied a range of water-miscible organic solvents, stabilizing agents, and the gelation conditions to minimize changes from occurring in the aerogel setting and the supercritical drying process. It has been found that atmospheric carbon dioxide has a significant effect on aggregation, and it cannot be entirely excluded under normal synthetic conditions. Methanol resulted in an increase in the particle size only, while dimethyl sulfoxide, dimethylformamide, and urea changed the shape of nanoparticles to rod-like shapes, and diols led to an increase in both size and shape. However, using the polymeric stabilizer poly(vinyl pyrrolidone) efficiently prevented the aggregation of the particles, even in the presence of high concentrations of carbon dioxide, and allowed the production of nanoAu containing silica aerogels in a single step, without the modification of technology.

## 1. Introduction

When dispersed down to the few nanometer sizes, gold is not a “noble” metal anymore. Nanogold particles (AuNPs) were extensively studied in the last two decades in several fields of science including physics, chemistry, biology, and medicine, leading to a sort of science “gold rush” [1]. Due to their high atomic number, electric properties, small size, and functionalizable surface, several practical applications have now been found for the gold nanoclusters. Plasmonic gold layers are embedded in surface plasmon resonance (SPR) detectors [2], and variable-sized injectable gold nanoparticles with or without porous or polymeric shells are used in biomedical or photoacoustic imaging [3,4], immune and stem cell tracking [5], X-ray CT and NIR imaging [6], and theragnostics [7]. Functionalized and core-shell type gold nanoparticles (NPs) are new candidates for targeted drug delivery [8].

Gold nanoparticles are most commonly synthesized from tetrachloroauric acid HAuCl_4_ under a reductive environment that is provided by reducing agents like citric acid or NaBH_4_ [9,10,11,12]. Most recently, liquid phase laser ablation is also used to provide gold nanoparticles in a chloride-free environment [13]. The prepared nanoparticles must be stabilized in the solution with sulfur-containing molecules, polymers, or proteins to prevent their aggregation [14,15].

AuNPs of less than 5 nm in size are chemically reactive [16] and act as heterogeneous-phase catalysts in a high number of synthetic chemical reactions like hydrogenation, selective oxidations, carbon-carbon bond formation, coupling, cyclization, and isomerization [17]. Gold nanoparticles that are larger than 10 nm play important roles in biomedical imaging and plasmonic applications [18,19,20].

Gold NPs are very important in chemical syntheses [21]. However, their recovery after the reactions may be difficult or even impossible. Their immobilization on carriers with large surface areas results in catalysts that can be recovered and re-used more easily, thus decreasing the production costs and saving valuable noble metal resources [22]. High porosity carrier materials like silica, zirconia, and titania are commonly used materials [23,24,25], and most recently, gold nanoparticles immobilized in silica aerogels were reported [26,27,28,29].

Aerogels are very low-density solids which keep the original structural characteristics of the wet gels that they are prepared from. They can be made of virtually any material that can be gelled [30,31,32]. The most frequently used and known aerogels are silica aerogels, synthesized i.e., from alkoxysilane reagents in base or acid-base catalyzed sol-gel processes, and dried under supercritical conditions [33,34,35,36].

The immobilization of gold NPs in silica aerogel offers the advantage of ease of handling and removal, as well as protection from aggregation by the steric hindrance of particle movements. Such aerogels can be used in several fields, including plasmonic sensing and nonlinear optical experiments, optical fiber detection, and catalytic reactions [26,37].

In this study, our aim was to synthesize nanogold-containing silica aerogel nanocomposites for gas sensing and catalytic applications. A solution of 10 nm gold nanoparticles stabilized with citrate ions, as well as 2 nm AuNPs stabilized with poly(vinyl alcohol) (PVA) was used as nanogold sources, respectively. Here, we report the difficulties that arose from the aggregation of the nanoparticles, the effects of solvents and environmental gases, and the use of stabilizing agents in order to prepare the required AuNP-silica aerogel composites.

## 2. Results and Discussion

**Figure 1:**
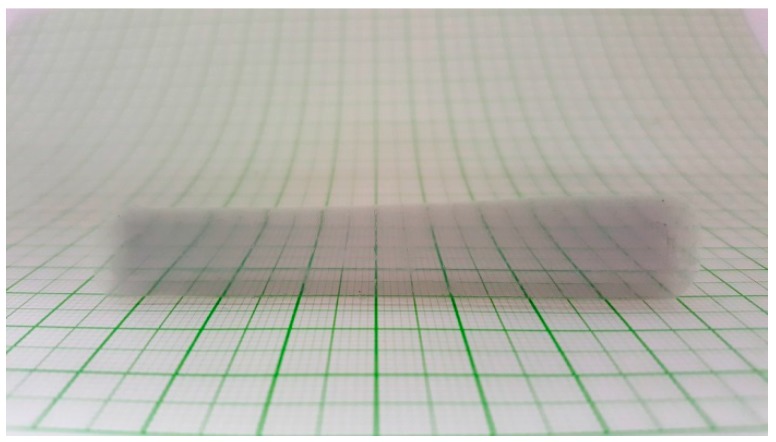
A photograph of a transparent base-catalyzed monolithic silica aerogel against a metric grid. The appearance of the grey color is the consequence of extensive aggregation of gold nanoparticles.

Silica aerogels were synthesized by the base-catalyzed sol-gel process, in which tetramethyl orthosilicate (TMOS) was hydrolyzed and condensed in a methanol-water mixture. In general, it is possible to mix particles or macromolecules in such a reaction mixture in order to create composites or hybrid aerogel materials, respectively [38,39]. However, when citrate-stabilized 10 nm Au nanoparticles were used as guest particles, a change of the original red color was observed in several experiments either during the hydrolysis or in the aging period, and the result was a grey-colored aerogel at the end of the process (Figure 1).

**Figure 2:**
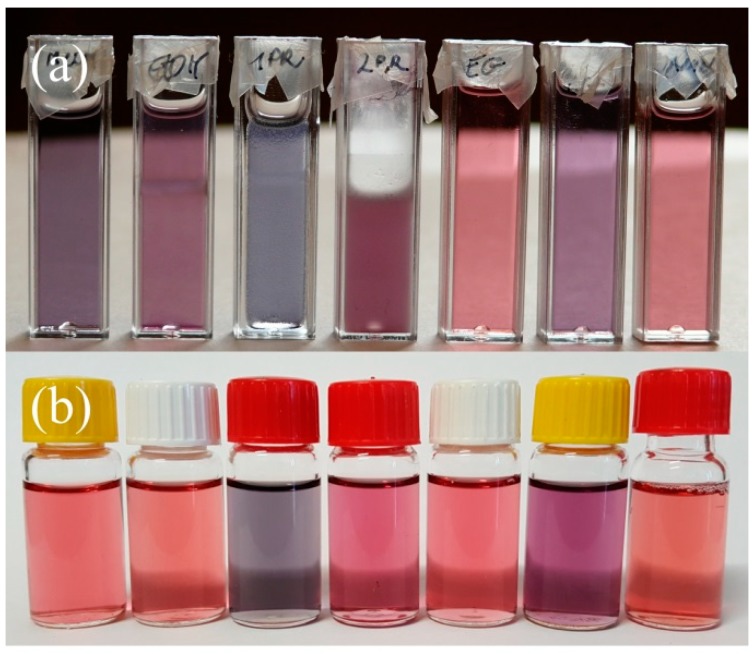
Photographs of the solutions containing 10 nm gold nanoparticles that were stabilized with citrate ions and diluted with an organic solvent (4 days of dilution): (**a**) in the parafilm-sealed cuvettes, very significant aggregation occurred, turning the solutions from red to grey or violet; (**b**) in hermetically the sealed sample vials, n-propanol and propylene glycol initiated strong aggregation, and the methanol solution remained unchanged.

The color change was due to the increased particle size of the AuNPs, as the plasmon resonance frequency is strongly correlated with the size and the shape of the nanogold particles [40]. In an attempt to prevent aggregation, we have tested different water-miscible solvents to find a composition in which AuNPs do not aggregate. Unfortunately, we found that the tested solutions behaved very differently when tested in sealed sample vials and when they were monitored spectrophotometrically. Figure 2 shows some photographs of the samples and the very significant difference in the aggregation of AuNPs between the parafilm-covered cuvettes and the hermetically sealed vessels. We have concluded that the most stable system contained diethanolamine (DEA) or ethylene glycol (EG) as the diluent. Their solutions were the least sensitive to the environmental effects. On the other side, n-propanol and propylene glycol (PG) were the solvents that initiated a rapid aggregation, even in sealed vessels. Methanol and ethanol are the most preferred solvents in our technology, however they behaved very differently in the cuvettes and in the sealed sample vials. This led to the conclusion that an atmospheric gas might also be responsible for the difference in aggregation.

**Figure 3:**
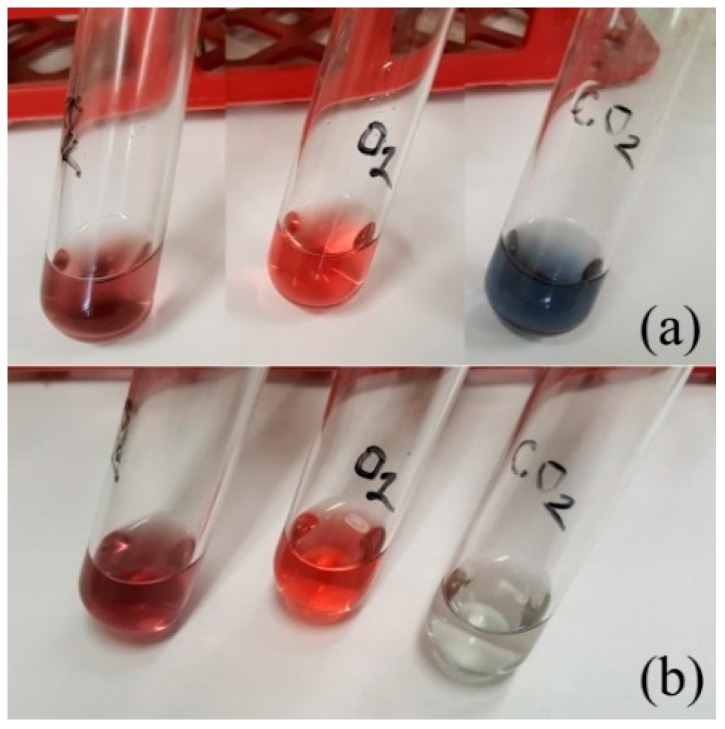
The effect of atmospheric gases on the stability of 10 nm Au sol in diethanol amine-containing solutions (from left to right: Ar, O_2_, CO_2_): (**a**) 15 min and (**b**) 4 days after being in contact with the given gas. Oxygen preserved the original color, and carbon dioxide led to aggregation in minutes and the settling of the micron-sized large particles in 4 days.

We have tested three atmospheric gases (Ar, O_2_, and CO_2_) for their aggregation-inducing power. Carefully vacuumed and argon-filled Schlenk-type glasswares were used in the studies, in which diethanolamine was used as the organic solvent. Since DEA can reversibly dissolve a significant amount of carbon dioxide, we heated it up to 100 °C and purged it with argon to remove all of the adsorbed gases before the experiments. After filling the vessels with the Au solutions, their gas phase was purged with the selected gas, and the vessels were then sealed hermetically. Figure 3 shows that in oxygen, no aggregation was initiated, even after 4 days. Carbon dioxide proved to be the most powerful aggregation agent and generated a strong color change in seconds. Surprisingly, under argon, some aggregation also took place. Considering that argon is a noble gas, this effect may be due to some other unknown factors. We isolated the dark grey precipitates from the solution and determined the particle sizes using optical microscopy (Figure S1). The diameter of the majority of the macroparticles were in the range of 0.8–3.0 µm. All showed a rounded shape near to spherical, indicating that the nanorod formation in the aggregation processes did not affect the shape of the aggregated macroparticles. The micron-sized particles were observed under a dark-field microscope and showed spectacular Brownian motion, as shown in Figure S2 and Video S1.

Although oxygen might seem to be a viable solution for the aggregation problem, we must consider that it would be extremely dangerous to use flammable materials under a pure oxygen atmosphere. In most laboratories, the use of that kind of mixture is strongly forbidden. On the other hand, by using our standard technology [34], it is not possible to provide a carbon dioxide-free environment, and thus another technique was needed to regain control over the process.

**Figure 4:**
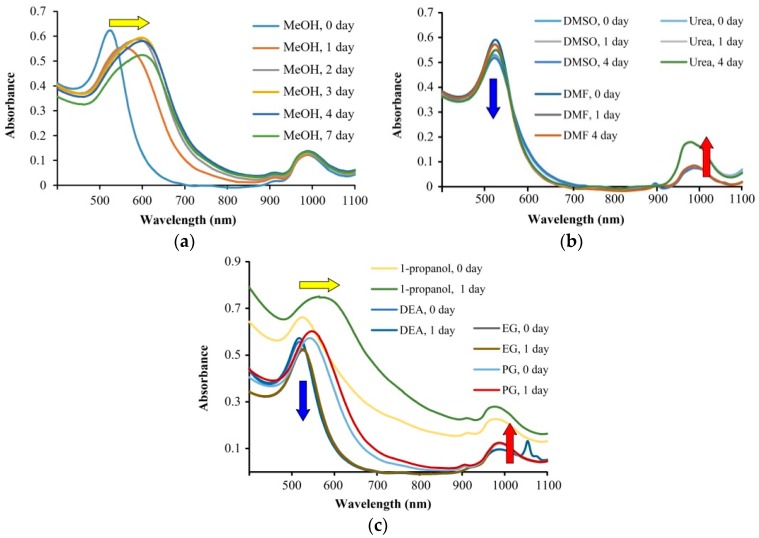
Spectral changes of nanogold solutions in 1–7 days after dissolution in different organic solvents. Depending on the nature of the solvent, spectral line intensity may be decreased (blue arrow) and increased (red arrow) or shifted to higher wavenumbers (yellow arrow). The spectral change corresponds to different types of aggregation: (**a**) the diameter of the spherical nanoparticles is increasing and the shape is preserved; (**b**) aggregation is occurring mainly in one dimension, resulting in the formation of rod-shaped particles; (**c**) the change of diameter and shape takes place simultaneously.

In order to determine what kind of aggregation was taking place in the different solvents, as well as to check the time-dependent behavior of the aggregation process, we have performed spectrophotometric studies with different organic solvents as diluents. The results are shown in Figure 4. Please note that the 10 nm AuNP stock solution showed a plasmon resonance peak at 980 nm, which was due to the presence of rod-like particles that were formed on storage under ambient conditions.

Three kinds of behavior were observed. In methanol (Figure 4a) a significant increase of spherical particle sizes occurred, which was indicated by the shift of the plasmon resonance peak from 520 nm to 620 nm. This corresponds to particles larger than 100 nm, which are calculated from the size-wavelength calibration curve published in the literature [36]. We monitored the spectra for 7 days in methanol, however we found that changes were insignificant after 2 days. Dimethyl sulfoxide (DMSO), dimethylformamide (DMF), and urea (Figure 4b) resulted in the formation of rod-like particles, which was indicated by the increase of the second plasmon resonance peak that was present at 980 nm, which is characteristic to the longer dimension of the particles. In the spectra, a decrease of peak intensity around 520 nm is associated with the increase of absorbance at 980 nm, as indicated by the blue and red arrows, respectively. The third group of solvents resulted in a simultaneous increase in size and the formation of larger nanorods, and their spectral changes were indicated by the yellow, blue, and red arrows in Figure 4c. To this group belongs 1-propanol, EG, PG, and DEA. DEA and EG are especially interesting because in the previous study, they seemed to be the most stable solvents. This indicates that even when no significant changes are visible to the naked eye, aggregation may happen in the nanogold solutions. The addition of an organic solvent changed the polarity of the AuNP solution significantly. Among the solvents, the most apolar n-propanol (logP_ow_ 0.34) resulted in the highest degree of aggregation.

We have tested different stabilizing agents to prevent the aggregation of the AuNPs in the aerogel reaction mixture. Poly(vinyl alcohol) (PVA), poly(ethylene glycol) (PEG), and poly(vinyl pyrrolidone) (PVP) were tested as polymeric agents. In addition, mercaptopropionic acid (MPA) and 3-mercaptopropyl trimethoxysilane (MPTMOS) were also tested due to the high affinity of gold NPs to sulfur donor atoms. PEG was sorted out in the process due to its low solubility in the TMOS hydrolysis mixture. For the stability tests, i-propanol, and EG was used as an aggregation-inducing solvent against which the stabilizing agents had to prove their power. MPTMOS was used in low concentration to create core-shell type particles. However, when mixed in the nanogold solution, it formed a precipitate in the solution. MPA was unable to prevent aggregation. PVA and PVP were both selected and tested in the real TMOS hydrolysis mixtures. Although they showed comparable activity, the dissolution of PVA in water took much longer, and it occasionally formed a precipitate in the reaction mixture with hydrolizing TMOS. As the stabilizing agent of choice, PVP proved to be the most advantageous among the tested ones, as it dissolved very rapidly, was compatible with the components of the reaction mixture, and prevented the aggregation of AuNPs until gelation occurred.

## 3. Conclusions

It has been found that the aggregation-free and reproducible synthesis of plasmonic silica aerogels containing small gold nanoparticles is a hard-to-control process. The system may behave differently, depending on the type of reaction vessel, the nature of the solvents used in the process, as well as the composition of the environmental gases. Atmospheric carbon dioxide has a strong negative impact, and solvents like n-propanol may initialize aggregation, even without carbon dioxide. After testing several stabilizing agents, poly(vinly pirrolidone) proved to be the most advantageous one. By using PVP in the TMOS-NH_3_-water-methanol mixtures, it was possible to reproducibly synthesize multi-centimeter sized monolithic silica aerogel-gold nanocomposites without any aggregation of the nanogold guest particles.

## 4. Materials and Methods

The following chemicals and reagents were purchased and used without further purification. 10 nm Nanogold solution stabilized with citrate (Sigma-Aldrich, Cat. No. 741957, St. Louis, MO, USA), 3-mercaptopropyl trimethoxysilane (MPTMOS) (Sigma-Aldrich Kft., Budapest, Hungary, Cat. No. 175617), Diethanolamine (Merck Kft., Cat. No. 769081, Budapest, Hungary), ammonia solution of 25% (Molar Chemicals, Halásztelek, Hungary, Cat. No. 00890-101-340), tetramethoxysilane (TMOS) (Fluka, 87682, St. Gallen, Switzerland), methanol (technical grade, Molar Chemicals, 05730-006-410), methanol (hplc grade, Sigma, Cat. No. 34860), acetone (99.98%, Molar Chemicals, Cat. No. 00620-006-410), 2-propanol (99.96%, Molar Chemicals, Cat. No. 00390-101-340), 1-propanol (99.92%, Molar Chemicals, Cat. No. 00200-006-410), propylene glycol (99.5%, Fluka, Cat. No. 82280), ethylene glycol (a.t., Reanal, Cat. No. 05039), ethanol (96.25%, Molar Chemicls, Cat. No. 02911-469-410), and poly(vinylpyrrolidone) (MW 40kD, Sigma-Aldrich, Cat. No. PVP40).

Photometry studies were performed with a Metertech SP-8001 spectrophotometer (Metertech Inc., Nangang, Taipei, Taiwan) in the 400–1100 nm range, using disposable PMMA cuvettes. Data were registered and analyzed by the Metertech UV-Mate software.

Alcogels were prepared in plastic molds, were aged, and solvent was exchanged in aluminum drying frames. Extractions and supercritical CO_2_ drying of alcogels to aerogels were performed in a custom–made high pressure reactor, according to the process described in the literature [34].

Aerogel SH1 was synthesized by the following method. First, two solutions (A and B) were prepared before the reaction. Solution A was made of 16.0 mL of MeOH and 4.00 mL of TMOS. Solution B contained 16.0 mL of MeOH, 3.60 mL of H_2_O, and 1.80 mL of aqueous ammonia solution (conc.: 25%). Solution A was magnetically stirred and solution B was mixed in rapidly. After 1 min of intensive stirring, the reaction mixture was poured into a 28 mm diameter cylindrical PVC plastic mold, which was lined with a thin poly(tetrafluoro ethylene) (PTFE) layer inside. The mold was covered with a double layer of parafilm to prevent evaporation. After one day of aging, the alcogel was transferred into a pitted aluminum frame and was soaked in methanol, methanol-acetone mixture, acetone, and copious amounts of freshly distilled dry acetone for 3 days to remove the water residues from the gel. After that, the wet gels were transferred into the high pressure tank reactor and were dried under supercritical conditions.

**Figure 5:**
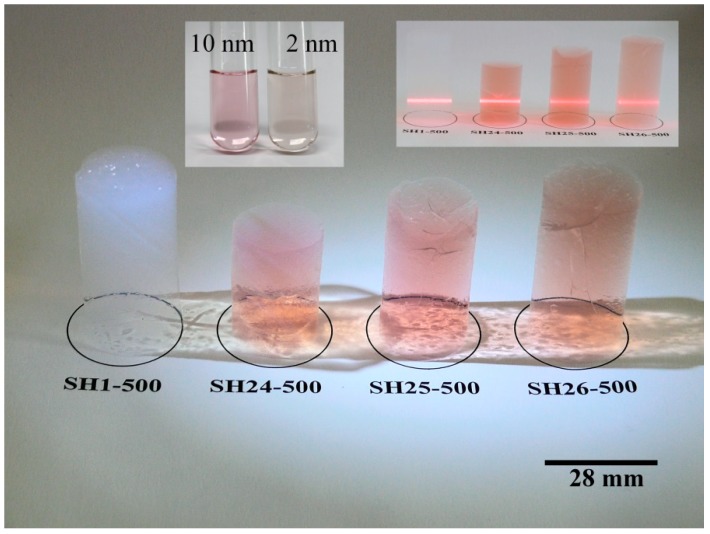
Photographs of native (SH1), as well as 10 nm (SH24, SH25) and 2 nm (SH26) gold nanoparticles containing monolithic silica aerogels after 500 °C/8 h of heat treatment. The top left insert shows the colors of the nanogold solutions in the same concentration as used in the aerogels. The top right insert shows the red laser light dispersion in the samples.

Aerogel SH24 was synthesized according to the following procedure: 3.00 mL of 10 nm gold solution and 2.40 mL of aqueous PVP solution (conc.: 10%) were combined, and then 15.0 mL of MeOH and 3.00 mL of TMOS were added. A 1:10 volume ratio dilution from a 25% ammonia solution was prepared, and 2.70 mL of that was added to the reaction mixture. The mixture was poured in a plastic mold, and then the process described for SH1 was followed. Aerogel SH25 was prepared by the method described for SH24 on a 50% larger scale. Aerogel SH26 was prepared by the method described for SH25, except that 2 nm PVA-stabilized nanogold solution was used. Photographs of the as-prepared aerogels SH1, and SH24-SH26 are shown in Figure 5.

### Sol Stability Studies

Water-miscible solvents that are compatible with the general sol-gel procedure were tested to compare their effect on nanogold particles’ aggregation. Two series of experiments were performed—one in open cuvettes, and the other in hermetically sealed sample vessels. Spectral changes were monitored and recorded in the first three hours, and then daily for five days. Each solution was a 1:1 volumetric dilution of the commercially available nanogold solution, with the solvent of choice.

## Supplementary Materials

The following are available online at http://www.mdpi.com/2310-2861/4/2/55/s1.
