# Supplementary material for: Prevention of the Aggregation of Nanoparticles during the Synthesis of Nanogold-Containing Silica Aerogels
Source: Gels. 2018 Jun 19;4(2):55. doi: 10.3390/gels4020055 (PMC6209257; doi:10.3390/gels4020055)
Supplement: Supplementary file 1 [file gels-04-00055-s001.zip › gels-315181-SI/Formatted suplementary.pdf]

gels

MDPI

Type of the Paper (Article, Review, Communication, etc.)

Suppementary materials

Prevention of aggregation of nanoparticles during the synthesis of nanogold-containing silica aerogels

István Lázár*, Hanna Judit Szabó

Department of Inorganic and Analytical Chemsitry, University of Debrecen, Egyetem tér 1, Debrecen, Hungary, H-4032

* Correspondence: lazar@science.unideb.hu; Tel.: +36-52-512900-22376

Received: date; Accepted: date; Published: date

Determination of particle sizes after precipitation occured in nanogold solutions

Grey particles formed in and settled out of nanogold solutions made with different solvents were isolated and examined under an Olympus bright field and a Zeiss dark field microscope. The images, as well as a video recording of the particle sizes, shapes and Brownian motion are shown in Figure 1, Figure 2 and Video S1, respectively.

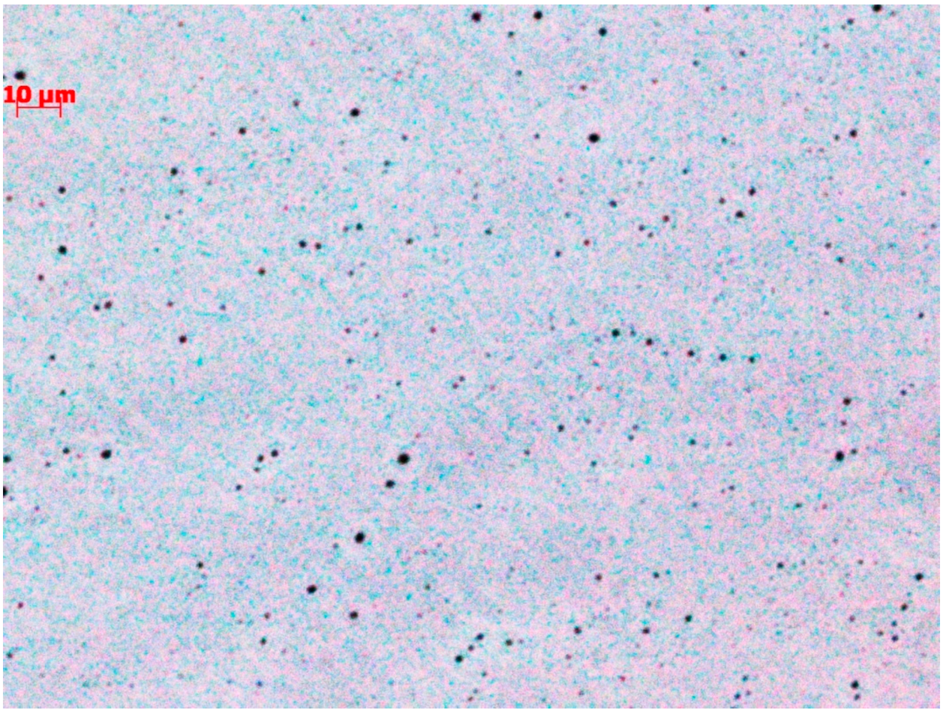

Figure S1. Optical microscopy image of settled micron-ranged gold particles isolated from carbon dioxide-treated nanogold solutions. A 10 µm scale bar is shown in red.

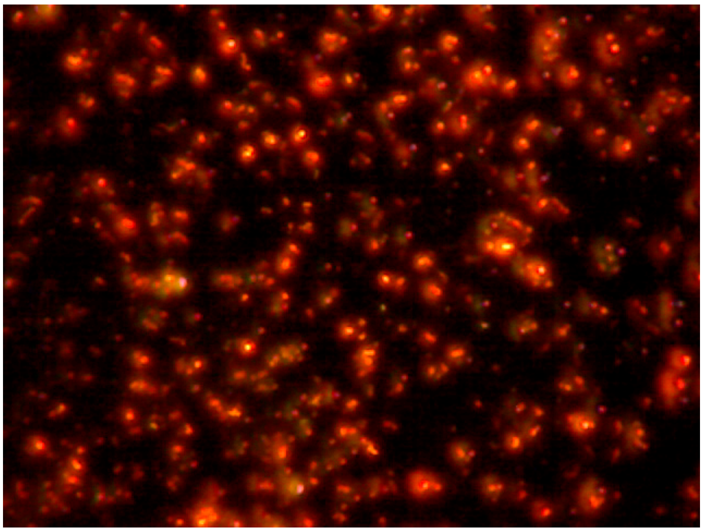

Figure S2. Dark field optical microscopy image of gold particles isolated from carbon dioxide-treated nanogold solutions (magn. 125 X). Picture was taken from Video S1 for static representation.

Video S1. Video recording of the Brownian motion of micron-sized gold particles observed with a dark field microscope equipped with a 2 MP digital eyepiece.

© 2018 by the authors. Submitted for possible open access publication under the terms and conditions of the Creative Commons Attribution (CC BY) license (http://creativecommons.org/licenses/by/4.0/).
