# Supplementary figures and images for: Prevention of the Aggregation of Nanoparticles during the Synthesis of Nanogold-Containing Silica Aerogels
Source: Gels. 2018 Jun 19;4(2):55. doi: 10.3390/gels4020055 (PMC6209257; doi:10.3390/gels4020055)

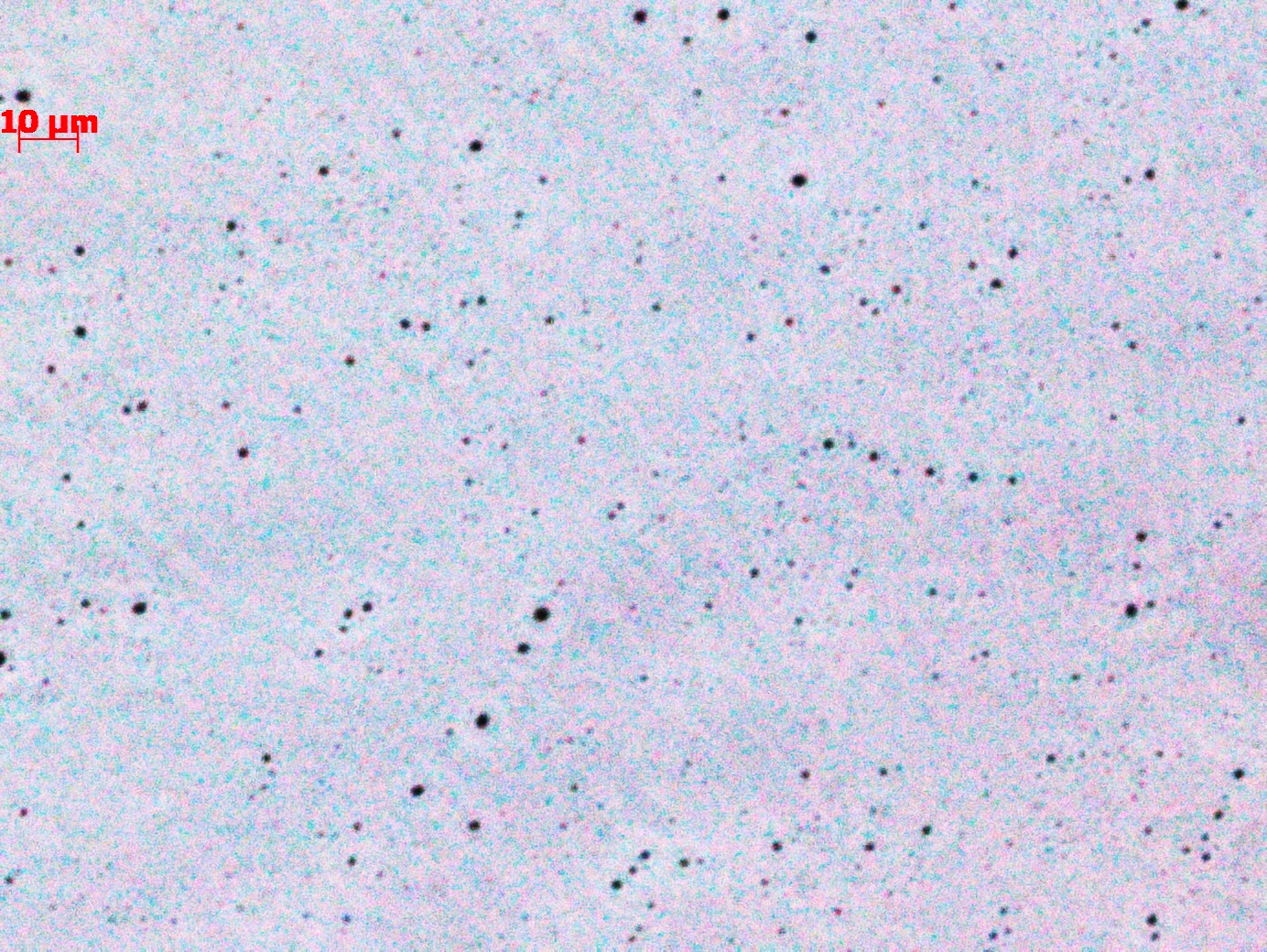

Supplement: Supplementary file 1 [file gels-04-00055-s001.zip › gels-315181-SI/Figure S1.jpg]

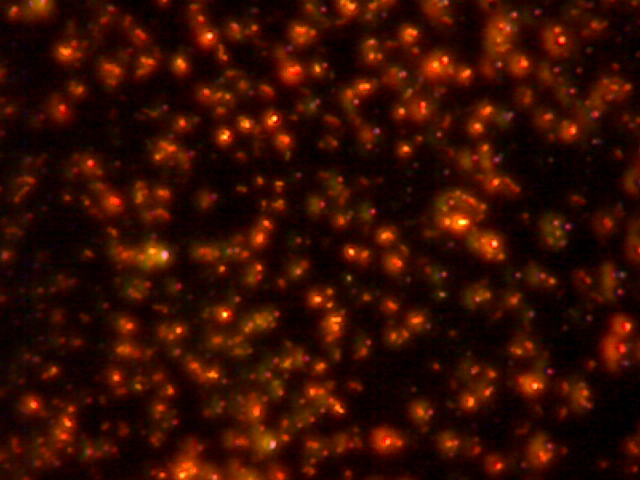

Supplement: Supplementary file 1 [file gels-04-00055-s001.zip › gels-315181-SI/Figure S2.png]
